# The RING-Finger Protein NbRFP1 Contributes to Regulating the Host Hypersensitive Response Induced by Oat Dwarf Virus RepA

**DOI:** 10.3390/ijms24097697

**Published:** 2023-04-22

**Authors:** Yanqing Liang, Zhanqi Wang, Qian Wang, Xueping Zhou, Yajuan Qian

**Affiliations:** 1 Institute of Biotechnology, Zhejiang University, Hangzhou 310058, China; 2 College of Life Science, Huzhou University, Huzhou 313000, China; 3 Department of Plant Protection, College of Agriculture and Biotechnology, Zhejiang University, Hangzhou 310058, China

**Keywords:** RING-finger protein, NbRFP1, hypersensitive response, RepA, oat dwarf virus, interaction

## Abstract

Our previous study identified that the RepA protein encoded by the oat dwarf virus (ODV) was responsible for inducing a strong hypersensitive response (HR) during the virus infection in non-host tobacco plants. However, little was known about the molecular mechanism of the RepA-elicited HR. Here, a RING-finger protein, which is described as NbRFP1 and is mainly located in the cytoplasm and nucleus in *Nicotiana benthamiana* cells, was confirmed to interact with RepA. In addition, the accumulation level of *NbRFP1* in *N. benthamiana* leaves was enhanced by either ODV infection or by only RepA expression. The knockdown of *NbRFP1* by a TRV-mediated virus-induced gene silencing markedly delayed the ODV or RepA-elicited HR. By contrast, the overexpression of *NbRFP1* in *N. benthamiana* conferred enhanced resistance to ODV infection and promoted RepA-induced HR. Further mutation analysis showed that a RING-finger domain located in NbRFP1 plays important roles in modulating RepA-induced HR, as well as in mediating the interaction between NbRFP1 and RepA.

## 1. Introduction

Plants often experience various biotic and abiotic stresses in natural ecosystems. In order to survive, plants have gradually formed multilayer defense mechanisms to adapt to various external stimuli [1]. PAMP-activated immunity (PTI), which is triggered by pathogen-associated molecular patterns (PAMP), and effector-activated immunity (ETI) are mainly represented in the plant defense portal [2]. The hypersensitive response (HR) is a typical manifestation of the ETI disease resistance, which is common in plant resistance responses to various pathogens, such as fungi, bacteria, viruses, and nematodes [3,4]. HR is usually associated with rapid cell death in the vicinity of pathogen primary infection, resulting in restricting pathogen spread [5]. The typical characteristics of HR include a burst of reactive oxygen species (ROS), the reprogramming of defense-related genes, the deposition of callose, and the activation of hormone signaling and the MAPK cascade [6,7,8,9,10].

Recent studies have shown that the ubiquitination-mediated 26S proteasome degradation system (UPS) plays an important role in the plant PTI-ETI defense system [11,12,13]. Protein ubiquitination is a common form of post-translational modification, which contains a series of cascade reactions catalyzed by the ubiquitin-activating enzyme (E1), ubiquitin-conjugating enzyme (E2), and ubiquitin ligase (E3) [14]. Currently, the identified E3 ubiquitin ligases mainly include two types: single-subunit E3 and multi-subunit E3 [15]. The single-subunit E3 is further divided into three types: HECT type, U-box type, and RING type. Meanwhile, the multi-subunit E3 mainly includes the SCF type, APC type, and VBC type [16].

The RING-type E3 ubiquitin ligase has a typical RING finger domain, which is necessary to maintain E3 ubiquitin ligase activity [17,18]. The RING domain contains 40–60 amino acids that bind the E2 in order to induce a closed E2-Ub conformation and to bind together the substrate [19]. Although many RING-type E3 proteins are found in plants, only some of them are reported to be involved in response to biotic stresses [20,21]. Emerging evidence has revealed that the pepper E3 ubiquitin ligase CaRING1 is induced by *Xanthomonas campestris* pv *vesicatoria* (XCV); furthermore, it serves in positive roles for HR production and disease resistance against XCV infection [22]. Similarly, rice OsBBI1, identified as RING E3 ligase, is induced by *Magnaporthe oryzae* (*M. oryzae*) and confers a broad resistance against *M. oryzae* [23]. In contrast, the type-III-secreted effector RipAC, which is obtained from *Ralstonia solanacearum*, was reported to interact with E3 ubiquitin ligase PUB4 in order to regulate kinase BIK1 homeostasis for the suppression of plant PTI immunity [24]. Furthermore, the *M. oryzae* avirulence effector AvrPiz-t was confirmed to target the RING E3 ubiquitin ligase APIP6 to promote the degradation of both proteins and to suppress PAMP-triggered immune responses in rice [25].

The oat dwarf virus (ODV) belongs to the genus *Mastrevirus* in the *Geminiviridae* family, which possess a circular single-stranded DNA genome with a full length of 2740 base pairs (bp) [26,27]. ODV encodes four proteins: a movement protein (MP or V1) and a coat protein (CP or V2) on the viral-sense strand, and two replication-associated proteins (RepA and Rep) on the complementary-sense strand [27]. Our previous reports revealed that the infection of ODV in non-host tobacco plants elicited a HR-like response. Subsequently, the ODV RepA protein was demonstrated to be responsible for HR-type cell death [9]. Further gene expression profiling that was produced by RNA-Seq confirmed 7878 significantly differentially expressed genes (DEGs) responses to the transient expression of ODV RepA, suggesting a complex and dynamic regulatory network involved in modulating RepA-induced HR [28].

Although ODV RepA had been manifested to trigger HR-like plant immunity, up to date, little was known about the host factor(s) that are involved in modulating RepA-induced HR. In this study, we identified that the RING-finger protein NbRFP1 is responsible for ODV infection and that it interacts with ODV RepA. An analysis of the knockdown and overexpression demonstrated that NbRFP1 positively regulates ODV RepA-induced HR. Additionally, the RING domain located in NbRFP1 serves important roles in regulating RepA-induced HR and the interaction with RepA. Our findings will provide new insights for a better understanding of the molecular mechanism of ODV RepA-induced HR.

## 2. Results

### 2.1. The Isolation and Sequence Analysis of NbRFP1

**Figure 1:**
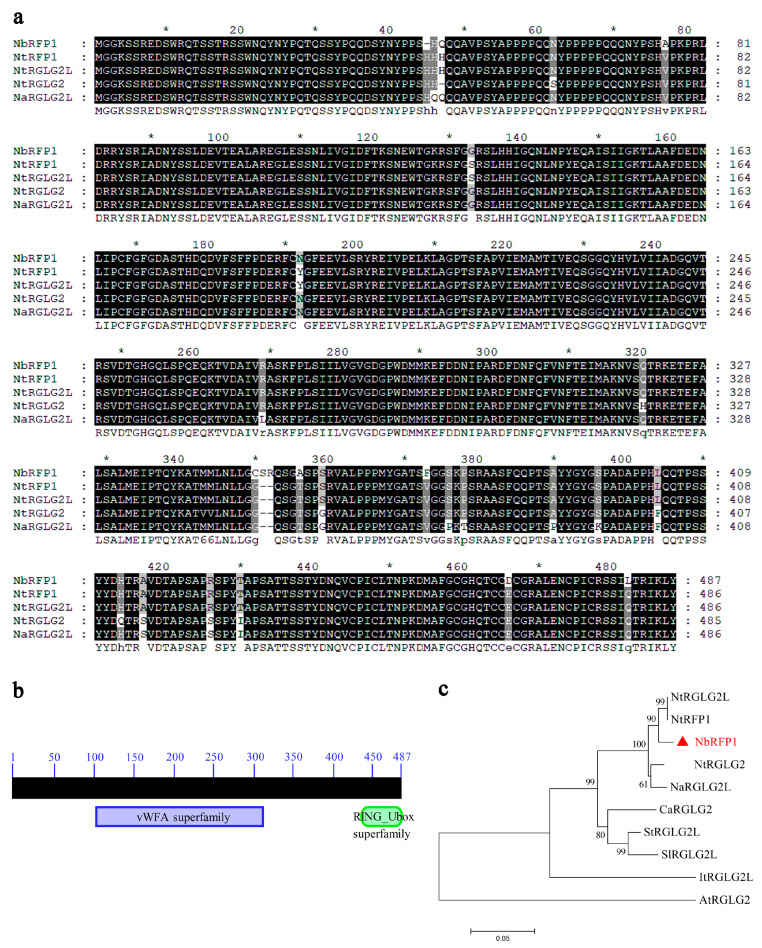
Bioinformatics analysis of NbRFP1. (**a**) The amino acid sequence alignment was carried out using MEGA 11 software and visualized using GeneDoc v2.7 software. (**b**) The conserved domain of NbRFP1 was predicted through the online InterPro. (**c**) The phylogenetic analysis based on the amino acid sequences derived from NbRFP1 and its homologs was performed using MEGA 11 software.

RING-typed E3 ligases are widely reported to be involved in plant defense responses. Previously, a tobacco RING-finger protein that was designated as NtRFP1 was demonstrated to be a functional E3 ubiquitin ligase and was found to mediate the degradation of geminivirus-encoded βC1 [17]. Here, a predicted RING-finger protein 1 (NbS00031234g0005.1) was cloned from the *N. benthamiana* cDNA library, where it is named *NbRFP1*. The *NbRFP1* coding region is 1464 bp in length and encodes a 487-amino-acid (aa) protein with an estimated molecular mass of 53.84 kDa (Figure 1a). A conserved domain search through the online InterPro (https://www.ebi.ac.uk/interpro/, accessed on 18 February 2022) revealed that the NbRFP1 protein contains a von Willebrand factor type A (vWFA) domain (which is located between 109–312 aa) and a RING-finger domain (spanning the region between 444–477 aa) (Figure 1b). A Blastx search, which was conducted using the complete coding sequence of NbRFP1 in NCBI, and further sequence alignments revealed that NbRFP1 shared the highest amino acid sequence similarity (98.1%) with *Nicotiana tabacum* NtRFP1 (GenBank accession: AGL95792) and the *N. tabacum* E3 ubiquitin-protein ligase RGLG2-like (NtRGLG2L) (GenBank accession: NP_001312603). In addition, phylogenetic analysis showed that NbRFP1 is more closely related to NtRFP1, NtRGLG2L, *N. tomentosiformis* E3 ubiquitin-protein ligase RGLG2 (NtRGLG2) (GenBank accession: XP_009599492), and *N. attenuata* E3 ubiquitin-protein ligase RGLG2-like (NaRGLG2L) (GenBank accession: XP_019223978) from *Nicotiana* plants (Figure 1c). These results suggest that NbRFP1 is a RING-finger protein that is closer to the NtRFP1 and NtRGLG2L from *N. tabacum*.

### 2.2. Expression Pattern and Subcellular Localization of NbRFP1

**Figure 2:**
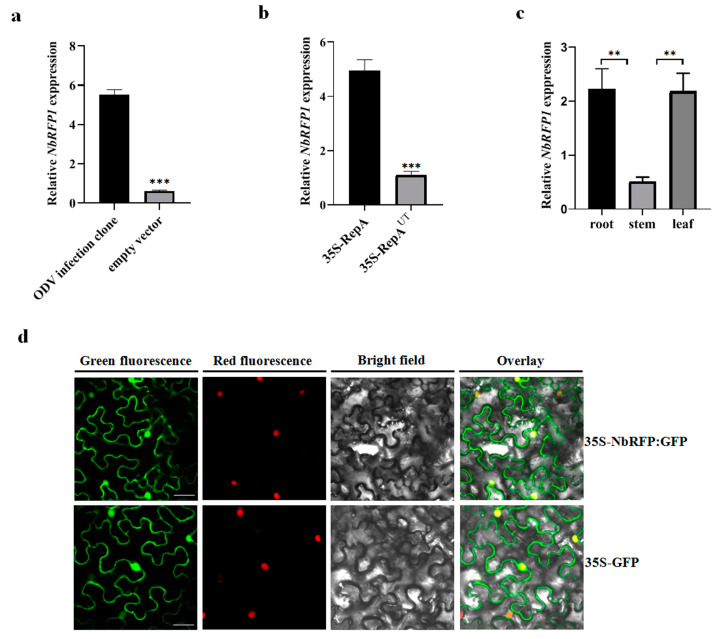
The expression pattern and subcellular localization of NbRFP1. (**a**,**b**) Reverse transcription-quantitative PCR (RT-qPCR) analysis of the expression levels of *NbRFP1* in the leaves of *N. benthamiana* following the ODV infection or transient expression of ODV RepA. 35S-RepA^UT^ served as an untranslatable mutant control. The data are given as the means ± the standard deviation (SD) of the three biological replicates. Asterisk indicated significant difference between treatments (** *p* ≤ 0.01; *** *p* ≤ 0.001). (**c**) The expression pattern of *NbRFP1* in the different tissues of *N. benthamiana* during the 5–6 leaf stage. Histone H3 was used as an internal reference. The data are given as the means ± the SD of the three biological replicates. (**d**) The sublocalization of the NbRFP1 in RFP-H2B transgenic *N. benthamiana* leaf epidermis. The GFP fluorescence was observed via confocal microscopy at 48 h post-infiltration (hpi). Histone 2B-RFP was used as a marker for the nucleus. Bar scale = 20 μm.

We evaluated the expression levels of *NbRFP1* in the leaves of *N. benthamiana* in response to ODV infection or the transient expression of ODV RepA. As shown in Figure 2a,b, when compared with the empty vector control, the expression of *NbRFP1* was significantly up-regulated by more than 5.0-fold and 4.0-fold following ODV infection and the transient expression of ODV RepA, respectively. These results suggest that both ODV and ODV RepA can positively regulate the transcript levels of *NbRFP1* in *N. benthamiana*.

To further investigate the biological functions of NbRFP1, we determined its tissue expression in *N. benthamiana*. As shown in Figure 2c, *NbRFP1* was ubiquitous in the examined roots, stems and leaves, displaying a similar expression pattern to that of the *NtRFP1* in *N. tabacum* [17]. Further analysis of RT-qPCR revealed the highest levels of *NbRFP1* transcripts were detected in roots and leaves at the 5–6 leave stage.

Subsequently, we examined the subcellular localization of NbRFP1 in leaf epidermis through the transient expression of the NbRFP1-GFP fusion protein in transgenic *N. benthamiana* overexpressing an RFP protein fused to a histone 2B (RFP-H2B) nuclear marker. The NbRFP1-GFP fluorescence was uniformly distributed in both the cytoplasm and the nucleus in the epidermal cells of *N. benthamiana* (Figure 2d), which was similar to that of the *N. benthamiana* that was infiltrated with 35S-GFP alone (Figure 2d). In order to further evaluate whether NbRFP1 is located in cytoplasm, PIP2A-mCherry, a marker for the plasma membrane, was co-agroinoculated with 35S-NbRFP1:GFP. The merged image revealed NbRFP1 was not closely associated with the plasma membrane (Figure S1). Taken together, these observations suggest that NbRFP1 is primarily localized in the cytoplasm and nucleus.

### 2.3. Silencing of NbRFP1 Delays ODV RepA-Induced HR

**Figure 3:**
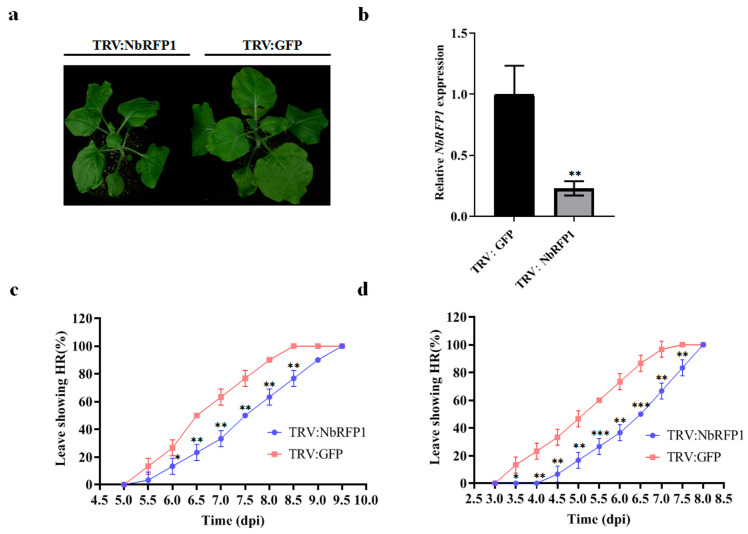
The effects of silencing *NbRFP1* on ODV or RepA-induced HR. (**a**,**b**) Phenotype in *NbRFP1*-silenced *N. benthamiana* plants (**a**) and the silencing efficiency (**b**) of *NbRFP1* through the TRV-mediated VIGS system. The photograph was taken and the silencing efficiency was examined at 10 dpi, and *N. benthamiana glyceraldehyde-3-phosphate dehydrogenase* (*NbGADPH*) was used as an internal reference gene for RT-qPCR analysis. The data represent the means ± the SD of the three biological replicates. (**c**,**d**) The percentage of exhibiting HR symptoms in the control (TRV:GFP) and *NbRFP1*-silenced *N. benthamiana* plants (TRV:NbRFP1) that were agroinfiltrated with ODV infectious clones (**c**) or with 35S-RepA (**d**) over time. The data represent the means ± the SD of the three biological replicates. Statistical analysis was performed using two-way ANOVA followed by Student’s *t* test, and significant difference between treatments was indicated by asterisk (* *p* ≤ 0.05; ** *p* ≤ 0.01; *** *p* ≤ 0.001).

Our previous study has shown that ODV RepA is responsible for eliciting HR-type cell death during ODV infection [9]. To investigate the role of NbRFP1 in the induction of HR caused by ODV infection or ODV RepA, we used the tobacco rattle virus (TRV)-based virus-induced gene silencing system to silence the endogenous *NbRFP1* in *N. benthamiana* seedlings (Figure S2). At 10 days post inoculation (dpi) with TRV-derived constructs (Figure 3a), systemic leaves were sampled and subjected to RT-qPCR analysis. Compared with the TRV1 and TRV2:GFP-infiltrated control plants, the transcript level of *NbRFP1* in plants co-agroinfiltrated with TRV1 and TRV2:NbRFP1 decreased by approximately 80% (Figure 3b). Afterward, the control and silenced plants were infiltrated with agrobacterium cultures containing 35S-RepA or the infectious clone of ODV, respectively. They were then monitored for symptom development over time. In *NbRFP1*-silenced *N. benthamiana* plants, 50% of the leaves inoculated with ODV infectious clone presented HR-like symptoms at 7.5 dpi, while all inoculated leaves showed cell death at 9.5 dpi. In contrast, in the TRV1 and TRV2:GFP-infiltrated control plants, approximately 50% of the leaves that were inoculated with ODV infectious clones showed cell death at 6.5 dpi and all inoculated leaves presented HR-like symptoms at 8.5 dpi (Figure 3c). Similarly, HR-like cell death caused by 35S-RepA in *NbRFP1*-silenced plants was severely delayed compared to the control plants (Figure 3d). These results suggest that the silencing of *NbRFP1* negatively regulates ODV RepA-elicited HR.

### 2.4. The Overexpression of NbRFP1 Enhances ODV RepA-Induced HR

**Figure 4:**
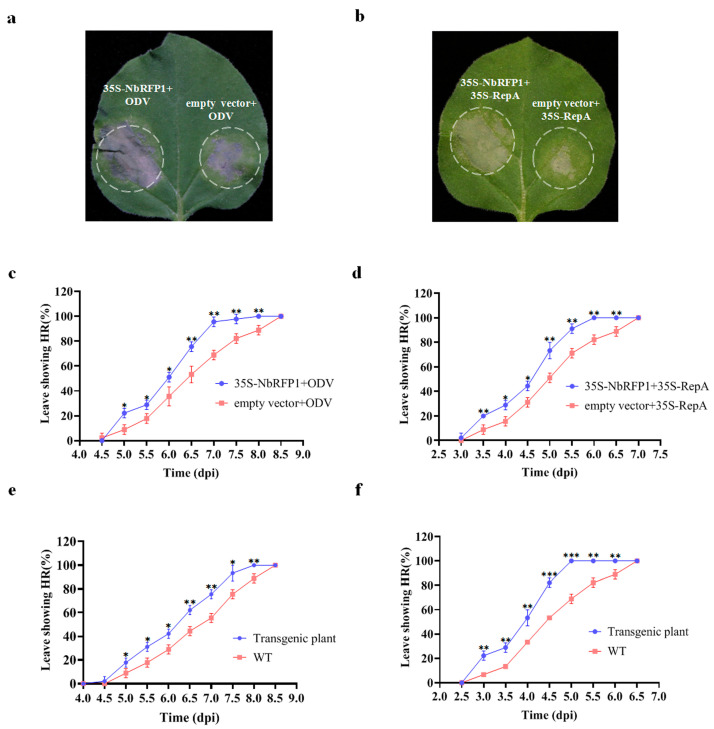
The effects of overexpression of *NbRFP1* on ODV RepA-induced HR. (**a**,**b**) Representative leaves in *N.benthamiana* plants agroinoculated using 35S-NbRFP1 and ODV infectious clones or 35S-RepA together. Photographs were taken at 3.5 dpi (**a**) or 6.0 dpi (**b**). (**c**,**d**) The percentage of exhibiting cell death in co-agroinfiltrated leaves using 35S-NbRFP1 and ODV infectious clones (**c**), or 35S-RepA (**d**) together. (**e**,**f**) The percentage of exhibiting cell death in wild-type leaves and *NbRFP1*-overexpressing transgenic *N. benthamiana* leaves agroinfiltrated with ODV infectious clones (**e**), or 35S-RepA (**f**). The data represent the means ± the SD of the three biological replicates. Statistical analysis was performed using two-way ANOVA followed by Student’s *t* test, and significant difference between treatments was indicated by asterisk (* *p* ≤ 0.05; ** *p* ≤ 0.01; *** *p* ≤ 0.001).

Since the down-regulated expression of *NbRFP1* in *N. benthamiana* plants by VIGS-mediated gene silencing could postpone HR induced by ODV or ODV RepA, we next determined whether the overexpression of NbRFP1 produced opposite effects on the production of RepA-elicited HR. As shown in Figure 4a,b, cell death elicited by ODV or RepA was produced faster while overexpressing NbRFP1. Further time-course observation identified 50% of the *N. benthamiana* leaves co-agroinfiltrated with 35S-NbRFP1 and ODV infectious clones demonstrated HR-like necrosis at approximately 6 dpi, while all infiltrated leaves showed cell death closely at 8 dpi (Figure 4c). Meanwhile, the control plants co-agroinfiltrated with empty vectors and ODV infectious clones showed HR-like symptoms with approximately a one-day delay, indicating that the transient overexpression of *NbRFP1* could promote the production of HR that are caused by ODV infectious clones (Figure 4c). Similarly, the results showed approximately 50% of *N. benthamiana* leaves co-agroinfiltrated with 35S-NbRFP1 and 35S-RepA demonstrated cell death at approximately 4.5 dpi, and all inoculated leaves showed HR symptoms at 6.0 dpi (Figure 4d). However, 50% of the *N. benthamiana* leaves were co-agroinfiltrated with empty vectors and 35S: RepA showed HR phenotypes until 5.0 dpi; all plants showed HR-like cell death at 7.0 dpi (Figure 4d). These results indicate that the transient overexpression of *NbRFP1* can significantly promote ODV RepA-induced HR.

To further clarify the role of *NbRFP1* in the induction of HR symptoms in *N. benthamiana*, we generated *NbRFP1*-overexpressing transgenic plants through agrobacterium-mediated genetic transformations. The positive transgenic plants were confirmed by Western blotting analysis and were used for further examination. When the transgenic plants were inoculated with ODV infectious clones, approximately 50% of the inoculated leaves showed HR at 6 dpi and 100% of the inoculated leaves showed a HR phenotype at 8 dpi. A significant positive shift of 0.5 day was observed when compared with the control plants (Figure 4e). Similarly, as shown in Figure 4f, approximately 50% of the transgenic *N. benthamiana* plants showed HR in their infiltrated leaves at 4 dpi after 35S-RepA inoculation; all inoculated leaves displayed HR symptoms at 5 dpi. In contrast, approximately 50% of the wild-type *N. benthamiana* showed HR symptoms until 4.5 dpi after 35S-RepA inoculation, and all inoculated plants displayed HR symptoms at 6.5 dpi (Figure 4f). Collectively, these results suggest that the overexpression of *NbRFP1* can remarkably promote ODV RepA-induced HR in *N. benthamiana*.

### 2.5. ODV RepA Interacts with NbRFP1

**Figure 5:**
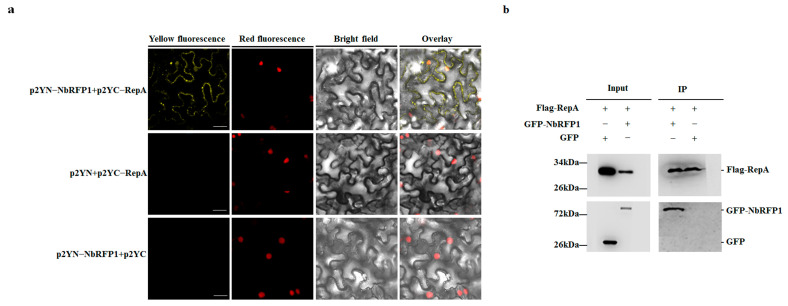
ODV RepA interacts with NbRFP1. (**a**) The interaction between RepA and NbRFP1 was examined using a BiFC assay in RFP-H2B transgenic *N. benthamiana* leaves, which were co-agroinfiltrated with the indicated combination. Fluorescence was observed via confocal microscopy at 48 h post-infiltration (hpi). Bar scale = 20 μm. (**b**) The interaction between RepA and NbRFP1 was examined using a CoIP assay. Leaves from different combinations were extracted before (input) and after immunoprecipitation (IP). Then, samples were individually analyzed by Western blotting using specific anti-Flag or anti-GFP antibodies.

To examine the interaction between ODV RepA and NbRFP1, bimolecular fluorescence complementation (BiFC) and coimmunoprecipitation (CoIP) assays were individually performed in *N. benthamiana*. The full-length sequence of RepA or NbRFP1 was inserted into a p2YC or p2YN vector. As anticipated, no fluorescence could be observed in the leaf epidermal cells of *N. benthamiana* that were co-agroinfiltrated with p2YN and p2YC:RepA, or with p2YN:NbRFP1 and p2YC, respectively (Figure 5a). In contrast, when p2YC:RepA and p2YN:NbRFP1 were co-expressed in the leaf epidermal cells of *N. benthamiana*, their noticeable YFP fluorescence was detected (Figure 5a), thus indicating that ODV RepA can interact with full-length NbRFP1 in vivo. The CoIP assay showed that RepA-Flag specifically pulled down the NbRFP1-GFP, and that RepA-Flag could not pull down GFP (Figure 5b), further indicating that RepA interacts with full-length NbRFP1 in vivo. Taken together, these results suggest that ODV RepA interacts with full-length NbRFP1 in vivo.

**Figure 6:**
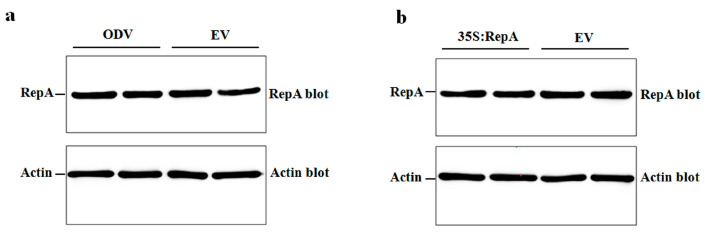
The assay of the stability of the ODV RepA protein in *NbRFP1*-overexpressing transgenic *N. benthamiana*. The detection of the accumulation levels of RepA in transgenic plants that were inoculated with ODV infectious clones (**a**) or 35S-RepA (**b**) by Western blotting with polyclonal antibodies that are specific for the RepA or Actin protein.

Next, we asked whether NbRFP1 affects the protein stability of ODV RepA in the process of RepA-induced HR. To this end, we examined the RepA protein abundance in control and *NbRFP1*-overexpressing transgenic *N. benthamiana* plants, which were inoculated with ODV infectious clones or 35S-RepA through Western blot analysis. As shown in Figure 6a,b, the overexpression of *NbRFP1* could not increase or decrease the ODV RepA protein abundance during ODV infection, nor could it affect the transient expression of 35S-RepA when compared to the controls. Together, these results suggest the interaction between NbRFP1 and RepA does not affect the stability of the ODV RepA protein.

### 2.6. The RING-Finger Domain in NbRFP1 Was Involved in the Interaction with RepA and Positively Regulated RepA-Induced HR

**Figure 7:**
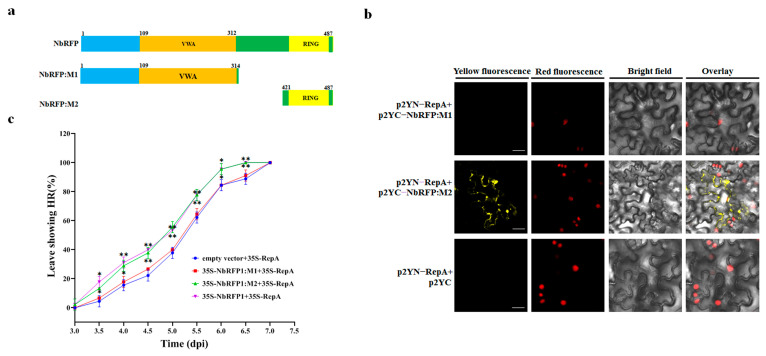
The assay of the effect of the mutation produced in NbRFP1 following the interaction with ODV-RepA and RepA-induced HR. (**a**) The schematic representation of the truncated mutants of NbRFP1. (**b**) The interaction between the mutants of NbRFP1 and RepA was examined using a BiFC assay in RFP-H2B transgenic *N. benthamiana* leaves. Bar scale = 20 μm. (**c**) The percentage of cell death in *N. benthamiana* leaves were co-agroinfiltrated with 35S-NbRFP1:M1 or 35S-NbRFP1:M2 together with 35S-RepA over time. The data represent the means ± the SD of the three biological replicates. Statistical analysis was performed using two-way ANOVA followed by Student’s *t* test, and significant difference between treatments was indicated by asterisk (* *p* ≤ 0.05; ** *p* ≤ 0.01).

In order to determine the domain of NbRFP1 that is involved in modulating the interaction with ODV RepA, we constructed truncated mutants of NbRFP1. As shown in Figure 7a, NbRFP1-M1 deleted 315 to 483 amino acids in the C-terminus, while NbRFP1-M2, which contains a RING-finger domain, deleted N-terminal 1 to 420 amino acids. In vivo BiFC assays indicated that the co-expression of p2YN-RepA and p2YC-NbRFP1:M2 produced yellow fluorescence signals, specifically mainly in the cytoplasm of tobacco cells, whereas the combinations of p2YN-RepA and p2YC-NbRFP1:M1 or p2YN-RepA and p2YC both failed to produce fluorescence signals (Figure 7b). These results suggest that the RING-finger domain may play an important role in modulating the interaction between NbRFP1 and RepA.

Previous reports revealed that the RING domain is necessary for E3 ligase enzyme activity, resulting in interference with the plant immune response [17,18]. To investigate whether the RING-finger domain can mediate RepA-induced HR-like cell death, the co-expression of the mutants of NbRFP1 and RepA were performed in *N. benthamiana* plants. As shown in Figure 7c, approximately 50% of the leaves were infiltrated with combinations of 35S-NbRFP1:M2 and 35S-RepA, or with 35S-NbRFP1 and 35S-RepA demonstrated cell death at 5 dpi and all infiltrated leaves showed HR phenotypes at 6.5 dpi. In contrast, a remarkable delay was observed in the leaves that were infiltrated with the combinations of 35S-NbRFP1:M1 and 35S-RepA or empty vectors and 35S-RepA (Figure 7c). These results suggest that the RING-finger domain might be involved in positively modulating ODV RepA-induced HR.

## 3. Discussion

Emerging reports confirm that particular pathogen proteins can elicit robust HR-type immune responses [29,30,31]. Previously, we have demonstrated that ODV RepA is an elicitor that induces HR during ODV infection [9]. However, little is known about the molecular mechanism regarding RepA-induced HR. In this study, we identified that the expression of *NbRFP1* was highly induced by ODV infection and was targeted by ODV RepA. A conserved domain search revealed that NbRFP1 contained a central conserved vWFA domain and a C-terminal RING-finger domain. Further sequence alignment revealed that NbRFP1 shared a 98.1% similarity with NtRFP1 from *N. tabacum*. Additionally, NtRFP1 has been confirmed as a RING-finger E3 ligase [17]. These cases of evidence strongly imply that NbRFP1 is a RING-type E3 ligase.

In plants, many RING-type E3 ligases have been widely reported to be involved in plant immune responses to abiotic and biotic stresses [32,33,34,35]. For example, Kim et al. reports the expression level of rice OsRFPHC-13, a RING-type E3 ligase, is sharply induced under salt stress. Accordingly, overexpression of OsRFPHC-13 in rice improves salinity resistance via ABA dependent manner [36]. Similar findings reveal the expression levels of Arabidopsis RING-type E3 ubiquitin ligases AtRDUF1 and AtRDUF2 are highly induced by flagellin 22 (flg22). Loss-of-function mutants of AtRDUF1 and AtRDUF2 result in inhibiting the PTI response elicited by flg22 and displaying susceptibility to *Pseudomonas syringae* (*P. syringae*) 3000 [37]. Evidence shows two closely related RPM1-interacting proteins, RIN2 and RIN3, that encode E3 ligase, and contribute positively to RPM1- and RPS2-dependent HR elicited by *P. syringae* strains expressing either the AvrRpm1 or AvrB type III effector proteins [38]. Recently, two rice RING-type E3 ligases, OsAPIP6 and OSPIP1, are discovered to positively regulate plant immunity by interacting with OSROD1 and reducing OSROD1 protein levels through UPS to interfere with Ca^2+^ sensor-mediated ROS scavenging [39]. ROD1 disruption results in broad-spectrum disease resistance to multiple pathogens, including *M. oryzae*, *Xanthomonas oryzae* pv. *oryzae* (Xoo), and Rhizoctonia solani (*R. solani*) [39]. In contrast, RIP1 or APIP6 knockout shows compromised resistance to *M. oryzae*, *Xoo*, and *R. solani* [39].

In line with these previous findings, our results demonstrate that NbRFP1 plays a positive role in regulating ODV RepA-elicited HR in *N. benthamiana*. The overexpression of *NbRFP1* could promote RepA-induced cell death, whereas the silencing of *NbRFP1* produced the opposite effect. It is worth noting that previous studies also revealed that the silencing of *NtRING1*, which is a RING-finger E3 ligase gene, can delay tobacco mosaic virus (TMV)-induced HR [40]. Several cases showed that an intact RING domain is essential for the enzyme activity of RING-type E3 ligases [17,41,42]. As expected, our investigation of the NbRFP1 mutants showed that the RING domain was required for NbRFP1 to positively regulate RepA-elicited HR.

In the past two decades, several effectors from fungi and bacterium have been reported to directly target RING-type E3 ligases [24,25,43]. Currently, several RING-finger E3 ligases have been demonstrated to positively interact with viral proteins in regulating viral infections. For example, OsRFPH2-10, a RING-Finger E3 ligase from rice, can interact and promote the degradation of the P2 that is encoded by the rice dwarf virus (RDV). In addition, it plays a critical antiviral function at the early stages of RDV infection in rice [44]. In *N. tabacum*, NtRFP1 could interact and mediate the degradation of the βC1 protein that is encoded by TYLVVNB (i.e., the satellite DNAβ that is associated with tomato yellow leaf curl China virus, TYLCCNB) resulting in the overexpression of NtRFP1, which attenuates viral symptoms [17]. It is well known that RING-type E3 ligase plays a crucial role in regulating plant immunity in various ways [12,45,46,47,48]. Recently, a microtubule-associated E3 ligase MEL from *N. benthamiana* and rice was reported to elicit a broad-spectrum host resistance to the rice stripe virus (RSV) and to a variety of other destructive pathogens by mediating the degradation of negative immune regulators [18]. In contrast, a functional RING-finger E3 ligase (AtRKP) was discovered to be induced by the C4 protein from the beet severe curly top virus (BSCTV), which played a negative role in the host resistance to BSCTV through degrading the protein ICK2/KRP2 and the mutation of AtRKP, which resulted in a reduced susceptibility to BSCTV [49].

It is widely acknowledged that many ubiquitin E3 ligases act as either positive or negative regulators of immunity by promoting the degradation of various substrates [12]. Here, our BiFC and Co-IP experiments demonstrated that NbRFP1 could function as a novel ODV RepA-interacting host factor. However, unlike the module of NtRFP1 and βC1, the analysis of the accumulation level of RepA in *NbRFP1* transgenic plants demonstrated that the overexpression of NbRFP1 could not affect the stability of RepA. Hence, it still remains to be addressed how the interaction of NbRFP1 and RepA contribute to RepA-induced HR. Interestingly, our BiFC experiments identified that the mutant of NbRFP1, with the deletion of the RING domain, failed to interact with RepA. The RING domain has been revealed to serve as a Ub-E2 docking site [16]. Recent reports have also revealed that Avr1d, an Avr effector from *Phytophthora sojae*, could act as an E2 competitor by occupying the site on GmPUB13 that is for binding the E2 ubiquitin conjugating enzyme [50]. Hence, this might suggest the possibility of RepA as an unknown E2 competitor by occupying its binding site. On the other hand, Li et al. discovered that the interaction between Avr1d and E3 ligase GmPUB15 is required for Avr1d recognition by R protein Rps1-b and Rps1-k [51]. Whether NbRFP1 mimics the similar modulating mode to be involved in regulating the plant immunity that is specific for RepA remains to be elucidated.

## 4. Materials and Methods

### 4.1. Plant Materials and Growth Conditions

Wild-type and *NbRFP1*-overexpressing transgenic *N. benthamiana* plants were used in this study. Transgenic plants with a constitutive expression of *NbRFP1* using a recombinant 3× Flag-pCAMBIA binary vector carrying the full coding sequence of *NbRFP1* were generated through agrobacterium-mediated genetic transformation. These plant materials were grown in a growth chamber at 25 ± 1 °C under a 16/8 h (light/dark) photoperiod and in 70 ± 5% relative humidity.

### 4.2. RNA Extraction, RT-PCR, and qPCR Analyses

Total RNA extraction from *N. benthamiana* leaves was performed using a TRIzol reagent (Invitrogen, Carlsbad, CA, USA), as was described previously [9]. The cDNA was reverse transcribed from 1 μg of the total RNA using a PrimeScript^TM^ RT Master Mix (TaKaRa, Shiga, Japan) according to the manufacturer’s instructions. RT-PCR and RT-qPCR analyses were carried out as described previously [9]. For RT-PCR analysis, the relative expression levels were calculated using a 2^−ΔΔCt^ method [52] and *N. benthamiana glyceraldehyde-3-phosphate dehydrogenase* (*NbGADPH*) was used as the internal reference.

### 4.3. Subcellular Localization

The full-length open reading frame (ORF) of *NbRFP1* was RT-PCR-amplified (the primers are listed in Table S1) from the *N. benthamiana* leaf’s cDNA and then introduced into the binary pCHF3 vector containing a GFP insertion to produce 35S-NbRFP1:GFP. The resulting 35S-NbRFP1:GFP and empty vector (35S-GFP) were electroporated individually into *A. tumefaciens* (C58C1). The transformed *A. tumefaciens* cultures were adjusted to an optical density at 600 nm (OD_600_) of 1.0 and infiltrated into leaves of four-leaf-stage RFP-H2B transgenic *N. benthamiana* seedlings. GFP fluorescence was observed and photographed using confocal microscopy (Leica TCS SP5, Leica, Mannheim, Germany) at 48 hpi.

### 4.4. BiFC Assay

The full-length ORFs of NbRFP1 and ODV RepA were cloned individually into p2YN and p2YC to produce p2YN-NbRFP1 and p2YC-ODV RepA. The truncated mutants of NbRFP1 were also inserted into p2YN to produce p2YN-NbRFP1:M1 p2YN-NbRFP1:M2. Additionally, the recombinant plasmids were then individually transformed into the *A. tumefaciens* (C58C1) and were co-agroinfiltrated into the leaves of four-leaf-stage *N. benthamiana* seedlings according to different inoculation combinations. The agroinfiltrated leaves were observed and photographed using confocal microscopy (Leica TCS SP5, Leica, Mannheim, Germany) at 48 hpi, as described by Li et al. [53].

### 4.5. Protein Extraction and Western Blotting Analysis

The total protein extraction from *N. benthamiana* leaves was performed using an extraction buffer, and the subsequently carried out Western blotting analysis was conducted as described previously [9]. The membranes were probed with specific polyclonal antibodies against Actin (ABclonal, Wuhan, China) or with specific polyclonal antibodies that were implemented against the ODV RepA produced by our lab.

### 4.6. CoIP Assay

The full-length ORFs of the ODV RepA were cloned into a 3×Flag-pCAMBIA vector to produce a Flag-RepA construct. Four-week-old *N. benthamiana* leaves were infiltrated, according to a 1.1 ratio, with *A. tumefaciens* containing Flag-RepA or GFP-NbRFP1 together. Then, the total protein was extracted at 2 days after agroinoculation with the IP buffer as described by Li et al. [54]. Immunoprecipitation was performed using an antibody against the Flag (α-Flag, Merck Sigma-Aldrich, Milwaukee, WI, USA). In addition, the immunoprecipitated proteins were analyzed by Western blotting analysis using an anti-GFP (α-GFP, Merck Sigma-Aldrich, Milwaukee, WI, USA) antibody. The immunoblot signals were detected as described previously [53].

### 4.7. Silencing of Endogenous NbRFP1

To induce the silencing of *NbRFP1* in the *N. benthamiana* plant, a TRV-based VIGS system was used. The 421 bp fragment from *NbRFP1* was cloned into a TRV-RNA2 vector. The *N. benthamiana* seedlings (5–6 leaf stage) were co-inoculated with equal amounts of agrobacterium cultures carrying TRV-RNA 1 or recombinant TRV-RNA2. At 10 dpi, the VIGS efficiency was evaluated by RT-qPCR using *GADPH* as the reference gene. Then, all silenced plants were subjected to HR assay through infiltration using agrobacterium cultures that contained 35S-RepA or ODV infectious clones. The cell death in the infiltrated leaves was observed over time.

### 4.8. Transient Co-Expression of RepA and NbRFP1

To transiently overexpress NbRFP1, or its derived mutant, the full ORF of NbRFP1 or the truncated mutants were individually cloned into the pCHF3 vector in order to generate the constructs of 35S-NbRFP1, 35S-NbRFP1:M1, and 35S-NbRFP1:M2. The co-expression of NbRFP1 and RepA, or the mutants of NbRFP1 and RepA by agrobacterium inoculation in *N. benthamiana* was carried out. Subsequently, the typical necrosis phenotype was observed over time.

### 4.9. Phylogenetic Analysis

Multiple sequence alignment was performed using MUSCLE (v3.8.31, a multiple sequence alignment method with reduced time and space complexity) with the default parameters [54]. The phylogenetic tree was constructed using the neighbor-joining (NJ) method via the use of MEGA11 software, and was evaluated using a bootstrap test with 1000 replicates.

### 4.10. Statistical Analysis

The data were shown as the means ± the standard deviation (SD) of the three independent biological replicates. Differences in the mean values were assessed using the Data Processing System (DPS, v15.10) [55], followed by the LSD test. The values were considered to be significantly different at a *p*-value of <0.05.

## 5. Conclusions

ODV RepA was previously reported to induce HR-type cell death during viral infection. However, little was known about the host factor(s) that are involved in modulating RepA-induced HR. In this study, our results indicate that the up-regulated abundance of NbRFP1 was identified in response to ODV infection or in ODV RepA transient expression. In addition, NbRFP1 plays a positive role in regulating RepA-induced HR by interacting with ODV RepA. To our knowledge, this is the first report of RING-type E3 ligase serving as a collaborator modulating ODV RepA-elicited HR. Our results will provide new insights for a better understanding of the molecular mechanism of the viral proteins involved in eliciting plant HR-type immune responses.

## Data Availability

The data presented in this study are available in this manuscript.

## Supplementary Materials

The supporting information can be downloaded at: https://www.mdpi.com/article/10.3390/ijms24097697/s1.
