# Supplementary material for: The RING-Finger Protein NbRFP1 Contributes to Regulating the Host Hypersensitive Response Induced by Oat Dwarf Virus RepA
Source: Int J Mol Sci. 2023 Apr 22;24(9):7697. doi: 10.3390/ijms24097697 (PMC10178201; doi:10.3390/ijms24097697)
Supplement: Supplementary file 1 [file ijms-24-07697-s001.zip › Supplementary files and figures.pdf]

Table S1: List of primers used in this study

Primer	sequences (5′- 3′)
Primers used for BiFC	
NbRFP1-p2YN- F	CCCTTAATTAACATGGGTGGAAAGAGTTCAAG
NbRFP1-p2YN- R	GGGACTAGTGTAGAGCTTTATTCGGGTTAG
NbRFP1:M1-p2YN-F	CCCTTAATTAACATGATGGGTGGAAAG
NbRFP1:M1-p2YN-R	GGGACTAGTGGCCATGATCTC
NbRFP1:M2-p2YN-F	CCCTTAATTAACATGCCATCTGCTCCC
NbRFP1:M2-p2YN-R	GGGACTAGTGTAGAGCTTTATTCG
Primers used for various expression vector	
RepA-Flag-pBA-F	GGTACCATGGCTACCGTTGCTTCC
RepA-Flag-pBA-R	GGATCCGAGATCTTGCCCAGGAAGTC
NbRFP1-pCHF3-F	GGTACCATGGGTGGAAAGAGTTCAAG
NbRFP1-pCHF3-R	GGATCCTCAGTAGAGCTTTATTCGGG
NbRFP1-pCHF3-NeGFP-R	GGATCCGTAGAGCTTTATTCGGGTTAG
Primers used for VIGS	
NbRFP1-TRV-RNA2-F	GAATTCCATCGGGCAGAATTTGAACC
NbRFP1-TRV-RNA2-R	GGATCCGTATAATCGACAAGGGAAACTT
Primers used for qRT-PCR	
NbGADPH-F	GCAGTGAACGACCCATTTATCTC
NbGADPH-R	AACCTTCTTGGCACCACCCT
NbRFP1-F	CATCGGGCAGAATTTGAACC
NbRFP1-R	GTATAATCGACAAGGGAAACTT

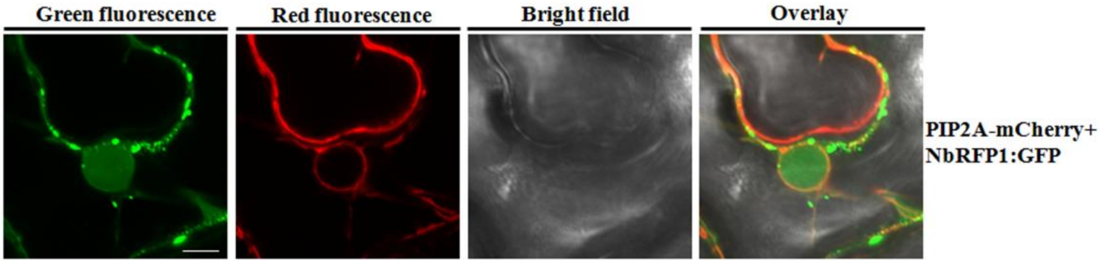

Figures S1: The sublocalization of the NbRFP1 in *N. benthamiana* leaf epidermis was assayed using PIP2A-mCherry as a marker for the plasma membrane. The GFP and RFP fluorescence was observed via confocal microscopy at 48 hpi. Bar scale = 10 μm.

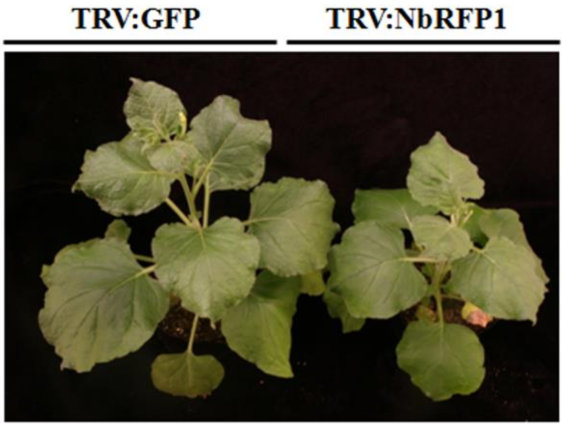

Figures S2: Photograph from *N.benthamiana* plants showing NbRFP1-silenced symptoms was taken at 28 days after co-agroinfiltration using TRV1 and TRV2:NbRFP1.
